# Income loss and subsequent poor psychological well-being among the Chinese population during the early COVID-19 pandemic

**DOI:** 10.1186/s12939-023-02022-1

**Published:** 2023-10-17

**Authors:** Sha Lai, Li Lu, Chi Shen, Alice Yan, Yanjun Lei, Zhongliang Zhou, Youfa Wang

**Affiliations:** 1 School of Public Policy and Administration, Xi’an Jiaotong University, Xi’an, China; 2 Division of Research Patient Care Services, Stanford Health Care, Palo Alto, CA 94305 USA; 3 Department of Pathogenic Microbiology and Immunology, School of Basic Medical Sciences, Xi’an Jiaotong University, Xi’an, China; 4 School of Public Health, Global Health Institute, Xi’an Jiaotong University Health Science Center, Xi’an, China

**Keywords:** Psychological well-being, Income loss, COVID-19, China

## Abstract

**Background:**

The COVID-19 pandemic has had major ramifications for health and the economy at both the individual and collective levels. This study examined exogenous negative changes in household income and their implications on psychological well-being (PWB) among the Chinese population during the COVID-19 pandemic.

**Methods:**

Data were drawn from the early China COVID-19 Survey, a cross-sectional anonymous online survey administered to the general population in China. Self-reported PWB was measured using a 5-point Likert scale with five questions related to the participants’ recent psychological state. Hierarchical multiple linear regression was employed to examine whether income loss during the COVID-19 pandemic was associated with poor psychological health.

**Results:**

This study included 8,428 adults, of which 90% had suffered from a moderate or severe loss of household income due to the early COVID-19 pandemic. Those who had experienced moderate or severe loss of income scored significantly lower on psychological well-being than those who did not experience income loss (19.96 or 18.07 vs. 21.46; P < 0.001); after controlling for confounders, income loss was negatively associated with PWB scores (moderate income loss: B = − 0.603, P < 0.001; severe income loss: B = − 1.261, P < 0.001). An interaction effect existed between the degree of income loss and pre-pandemic income groups. Specifically, participants in the middle-income group who had suffered severe income loss scored the lowest on PWB (B = − 1.529, P < 0.001). There was also a main effect on income loss, such that participants with varying degrees of income loss differed across five dimensions, including anhedonia, sleep problems, irritability or anger, difficulty with concentration, and repeated disturbing dreams related to COVID-19.

**Conclusions:**

Income loss during the pandemic has had detrimental consequences on psychological well-being, and the magnitude of the impact of income loss on psychological well-being varied according to previous income levels. Future policy efforts should be directed toward improving the psychological well-being of the economically vulnerable and helping them recover from lost income in the shortest time possible.

**Supplementary Information:**

The online version contains supplementary material available at 10.1186/s12939-023-02022-1.

## Introduction

Coronavirus disease 2019 (COVID-19), caused by the SARS-CoV-2 virus, was declared a pandemic by the World Health Organization on March 11, 2020. While preventive measures during the early stages of the pandemic were effective in reducing transmission, their economic costs were overwhelming [1–3]. Early COVID-19 pandemic mitigation measures pushed a large proportion of the human population into poverty, even extreme poverty [4]. Several surveys conducted during the early stages of the COVID-19 pandemic revealed a significant proportion of respondents who experienced varying degrees of income loss [5–9]. For instance, approximately 19.0% of adult respondents in the United States reported a decline in their income [5]. Similarly, in Germany, two-thirds of self-employed individuals witnessed a decrease in sales by at least 50% [6]. Among Spanish workers, 42.5% of respondents reported losing their incomes due to the pandemic [7]. In Israeli, 18.7% of adult respondents experienced a reduction in their income [5]. In Argentine, 51.7% of surveyed workers suffered financial losses [8]. Additionally, a significant proportion of Thailand’s population (49.0%) who were previously engaged in full-time employment experienced adverse economic impacts during the pandemic [9]. The diverse range of interventions implemented by various nations to mitigate the transmission of COVID-19 has resulted in varying economic consequence Overall, during the early stages of the COVID-19 pandemic, people suffered from both health shocks and income losses.

The COVID-19 pandemic has significantly affected public physical health, especially among those who experienced COVID-19 infection [10]. Moreover, an increasing number of studies indicate that the pandemic has had detrimental effects on mental health across countries with different levels of economic wealth, different pandemic response measures, and different quarantine requirements, both among the general population and within different subpopulations [11–16]. COVID-19 pandemic transmission reduction efforts have increased barriers to healthcare access and created an environment where many contributors to poor mental health were amplified. People exposed to COVID-19 and the resulting containment measures were placed under unprecedented pressure and experienced severe psychological distress [17]. For example, a large-scale meta-analysis from 32 countries revealed that about 25% of the population experienced stress, anxiety or depression symptoms, while nearly 75% of the population reported experiencing sleep problems during the early stages of the COVID‐19 pandemic [13].

Economic stress is a major risk factor for mental health issues [18]. Previous research indicates that loss of income may increase feelings of insecurity, shame, and stress [19]; raise the threshold for accessing mental health care services [20]; and negatively affect health and mortality [21, 22]. The correlation between financial stress, income loss, job loss and poor mental health during the pandemic is documented [5–9, 23–26]. For example, a national study in the USA revealed that individuals with less economic resources and greater exposure to unemployment stress reported a greater burden of depressive symptoms [27]. Another large study in the UK showed that people who experienced unemployment or had no source of income during the pandemic were more likely to be depressed than those who were gainfully employed [28]. A survey conducted at the same time in Spain found that household or individual income loss was associated with depression and panic attacks, and that perceived financial stress mediated this relationship [7]. In a study conducted by Liu et al., 398 Chinese respondents experienced income loss due to COVID-19 exhibited symptoms of depression (45.5%), anxiety (49.5%), insomnia (30.9%), and distress (68.1%) [29]. Collectively, these results highlight the importance of finance-related factors in personal mental health and psychological well-being affected by the COVID-19 pandemic.

However, the potential consequences of a decrease in income on the psychological well-being of individuals with varying pre-pandemic family economic statuses may differ. A comprehensive examination of the effects of income on health revealed that alterations in family income and the position of the household within the overall income distribution are significant indicators of well-being, and the influence of income fluctuations on well-being was not consistent across different income groups [30]. More specifically, fluctuations in disposable real income within households have a more pronounced impact on those with lower incomes [30]. According to Sturgeon et al., the impact of financial stress may vary based on social class, with individuals of higher social status perceiving financial stressors as particularly menacing due to the potential threat they pose to their identity as possessing a comparative advantage in resources over others [31]. While existing studies point to a link between the decrease in financial security related to COVID-19 and deterioration in mental health, the unique role of financial concerns in predicting mental health issues, over and beyond other pandemic-related concerns, has not been well-examined. Specifically, we examined two sources of variation in financial concerns: the degree of exogenous negative changes in one’s income and one’s past-year level of income.

In the early stages of the COVID-19 pandemic, the Chinese government adopted stringent non-pharmaceutical interventions to mitigate the spread of infection and reduce the burden of COVID-19 on healthcare systems, including a variety of containment measures, mass lockdowns, and remote work arrangements. According to our web-based survey, nearly 90% of respondents suffered from a moderate or severe loss of income due to the COVID-19 pandemic. The COVID-19 pandemic has continued for over three years, and the infection spread has come in waves in China. There are growing concerns regarding the influence of COVID-19-related income loss on psychological well-being and the development of mental health problems [32]. Due to the unique characteristics of the pandemic and the unique prevention and control measures in China, findings of other countries or regions may be difficult to transfer [7]. However, there have been no studies assessing the association between income loss and psychological well-being in the context of a large-scale infectious public health event in China. There is also limited understanding of the extent to which psychological well-being deteriorates as a function of income loss among the general population. The effect of a large-scale infectious public health event and its related containment strategies on individuals’ psychological well-being remains under-researched [24, 33, 34].

Natural experiments that produce exogenous changes in income have rarely been observed, making the question of establishing a causal relationship between income and PWB a major challenge [35]. The COVID-19 pandemic provides a unique opportunity to study the effects of income loss on psychological well-being. Since the source of such an economic impact is mostly exogenous and households who remained in employment also experienced income loss which mainly due to the COVID-19 pandemic [7]. Using data from the China COVID-19 Survey, we aimed to examine the following questions: (1) the relation between exogenous negative changes in household income and psychological well-being during the pandemic; (2) whether pre-pandemic levels of income moderated the relation between income loss and psychological well-being.

## Methods and materials

### Study design and data sources

The China COVID-19 Survey is a cross-sectional online survey conducted between April 25 and May 11, 2020. It was administered via WeChat, a cellphone application for communication used by more than a billion people in China [36–38]. Both snowball and convenience sampling approaches were employed to recruit a diverse sample in China. At the time this survey was initiated, there were more than 80,000 people infected with COVID-19 and 4,633 COVID-19-related deaths in China. The pandemic was generally sporadic, and clustered outbreaks caused by sporadic cases occurred in some areas. The corresponding prevention and control measures in most provinces in China have been downgraded from emergency response to a normalized management. Social isolation, lockdown, and travel restrictions were determined and implemented based on regional risk classifications.

The survey includes a national sample of 10,545 adults aged 18 years or above in China and 8,428 adults with complete data were analyzed in this study. The survey was voluntarily and anonymously completed. All subjects gave informed consent before they participated in the survey, and the protocol was approved by the Ethics Committee of Xian Jiaotong University (No.2020 − 1172).

### Measurements

Structured questionnaires were used to collect information on sociodemographic and economic characteristics, attitudes and behaviors towards COVID-19, general health conditions, chronic medical conditions, psychological well-being, and lifestyle habits [36].

#### Outcome variables

Psychological well-being (PWB) was assessed using a scale consisting of five items derived from the widely used and validated civilian version of the posttraumatic stress disorder checklist [39]. The participants were asked about the degree to which they experienced the following symptoms: (1) anhedonia, (2) sleep problems, (3) irritability or anger, (4) difficulty with concentration, and (5) repeated disturbing dreams related to COVID-19. The questions were as follows: “During the past month, have you experienced any of the following problems? To what extent did these problems bother you? 1): Lost interest in physical and social activities you liked in the past; 2): Difficulty falling asleep, or staying asleep, or waking up frequently, or early; 3): Got irritable or angry easily; 4): Difficulty with concentration; 5): Repeated disturbing dreams related to COVID-19.” Each item was rated on a 5-point Likert scale with the following response options: not at all = 5, a little = 4, some = 3, a lot = 2, extremely = 1. Total PWB scores ranged from 5 to 25, with lower scores reflecting poorer psychological well-being. The Cronbach’s alpha of this scale for our study was 0.912.

#### Independent variables

The primary independent variable of interest is the degree of income loss, which was measured using a self-reported question, i.e., “How has your family’s income been affected due to COVID-19?” The response options were “no income loss”, “moderate loss of income”, and “severe loss of income”.

We included demographic and socioeconomic characteristics, including gender (male/female), age (18–44/45–59/60 years or above), marital status (single/married or co-habiting), employment status at the time of filling out the questionnaire (employed/unemployed/non-employed, i.e., adult students and retired workers), education level (elementary school or below/junior or senior high school/bachelor’s degree and above), residential areas (city/town/rural areas), perceived risk of COVID-19 infection and actual risk level (low/medium/high) in respondent’s place of residence. The income gradient in the last year (i.e., before the pandemic) was divided into tertiles (low = 1st tertile/ middle = 2nd tertile/ high = 3rd tertile) according to the self-reported per capita household income in 2019. Health conditions included chronic medical conditions, self-rated health status, and whether participants or their family had experienced COVID-19 infection. The impact of COVID-19 on dietary patterns was assessed by asking participants: “To what extent has your diet been affected during the COVID-19 epidemic?” (no/general/high impact).

### Statistical analysis

We obtained basic descriptive statistics such as frequencies (n) and percentages (%) or means and their 95% confidence intervals (95% CI). We then conducted hierarchical multiple linear regression to examine whether income loss during the COVID-19 pandemic was associated with poor PWB. In step 1, the following predictor variables were introduced into the model for PWB: demographic and socioeconomic variables (age, gender, marital status, employment status, educational level, residential area and income level in the last year), health condition (chronic medical condition, self-rated health, participants themselves or their family members with confirmed COVID-19 infection) and health risk status (impact on diet caused by COVID-19, perceived risk of infection and actual risk level in the respondent’s place of residence), which were selected based on the knowledge of existing topic-related literature [40–44]. We also performed univariate linear regression analyses to evaluate potential confounders (all showed P < 0.05, in addition to residential area variables, see supplemental Table 1). In step 2, degree of income loss was added to the model. In step 3, we added the interaction term of income loss due to COVID-19 and income level in the past year. We used the R-square change (ΔR^2^) to assess the predictive power of each group of predictors after adjustments were made for predictors added in an earlier step of the model. Simple slope analyses were performed separately for the low, middle, and high level of income groups to further visualize the nature of the moderation.

To exclude reverse-causation bias between household income loss and poor PWB, we conducted sensitivity analyses within specific subgroups, namely non-employed individuals such as adult students and retired workers, as well as adult students. For non-employed individuals with fixed retirement pension or without income, especially students without income, poor mental health has little effect on family income loss. These analyses were carried out using hierarchical multiple linear regressions.

All statistical analyses were performed using STATA 14.0 (Stata Corporation, College Station, TX, USA). P < 0.05 (two-tailed) was considered to be statistically significant.

## Results

### Basic characteristics

Table 1 presents a summary of sample characteristics among participants who had completed “The 2020 China COVID 19 Survey”, by the level of COVID-19-related income loss. Referring to Table 1, 4, 207 (49.92%) participants reported having experienced moderate income loss, and 3,299 (39.14%) participants reported having experienced severe income loss. The differences in demographic and socioeconomic characteristics, health condition and health risk status among the three income loss subgroups were all statistically significant (P < 0.05).

**Table 1:** Sample characteristics among participants attending “The 2020 China COVID 19 Survey”, by the level of income loss due to the COVID-19

Variables		Whole sample (N = 8428)		Level of income loss						
				No (n = 922; 10.93%)		Moderate (n = 4207; 49.92%)		Severe (n = 3299; 39.14%)		χ^2^
		N	%	N	%	N	%	N	%	
Age	18–44^†^	7,387	87.7	742	80.5	3,631	86.3	3,014	91.4	101.19***
	45–59	945	11.2	155	16.8	529	12.6	261	7.9	
	60 or above	96	1.1	25	2.7	47	1.1	24	0.7	
Gender	Male^†^	3,694	43.8	351	38.1	1,692	40.2	1,651	50.1	86.48***
	Female	4,734	56.2	571	61.9	2,515	59.8	1,648	50.0	
Marital Status	Single^†^	2,617	31.7	245	26.6	1,319	31.4	1,107	33.6	16.68***
	Married or co-habiting	5,757	68.3	677	73.4	2,888	68.7	2,192	66.4	
Income level	Low	3,587	42.6	362	39.3	1,833	43.6	1,392	42.2	81.31***
	Middle	2,149	25.5	292	31.7	1,154	27.4	703	21.3	
	High^†^	2,692	31.9	268	29.1	1,220	29.0	1,204	36.5	
Employment status	employed^†^	5,768	68.4	668	72.5	2,981	70.9	2,119	64.2	115.59***
	unemployed	934	11.1	59	6.4	362	8.6	513	15.6	
	Non-employed	1,726	20.5	195	21.2	864	20.5	667	20.2	
Education level	Elementary school or below^†^	160	1.9	13	1.4	61	1.5	86	2.6	70.29***
	Junior high /High school	3,468	41.2	319	34.6	1,648	39.2	1,501	45.5	
	Bachelor’s degree or above	4,800	57.0	590	64.0	2,498	59.4	1,712	51.9	
Chronic medical condition	No chronic disease^†^	6,644	78.8	744	80.7	3,497	83.1	2,403	72.8	147.34***
	Having one chronic disease	845	10.0	96	10.4	379	9.0	370	11.2	
	multimorbidity	939	11.1	82	8.9	331	7.9	526	15.9	
Self-rated health	Very good^†^	6,859	81.4	748	81.1	3,410	81.1	2,701	81.9	12.65**
	good	1,336	15.9	155	16.8	695	16.5	486	14.7	
	Fair or poor	233	2.8	19	2.1	102	2.4	112	3.4	
COVID-19 infection (participants or family member)	Yes^†^	785	9.3	62	6.7	160	3.8	563	17.1	393.27***
	No	7,643	90.7	860	93.3	4,047	96.2	2,736	82.9	
Impact on diet	No impact^†^	726	8.6	252	27.3	321	7.6	153	4.6	1000.00***
	General impact	3,572	42.4	481	52.2	2,115	50.3	976	29.6	
	High impact	4,130	49.0	189	20.5	1,771	42.1	2,170	65.8	
Perceived risks of infection	Low^†^	3,787	44.9	628	68.1	2,132	50.7	1,027	31.1	1100.00***
	medium	3,065	36.4	221	24.0	1,711	40.7	1,133	34.3	
	high	1,576	18.7	73	7.9	364	8.7	1,139	34.5	
Risk level of living area	Low^†^	1,652	19.6	184	20.0	838	19.9	630	19.1	24.89***
	Medium	3,241	38.5	359	38.9	1,702	40.5	1,180	35.8	
	High	3,535	41.9	379	41.1	1,667	39.6	1,489	45.1	
Residential areas	City^†^	5,085	60.3	627	68.0	2,465	58.6	1,993	60.4	60.83***
	Town	2,067	24.5	198	21.5	1,140	27.1	729	22.1	
	Rural	1,276	15.1	97	10.5	602	14.3	577	17.5	

Note: † Reference levels in the regression

Chi-square test is used for balance checking between three groups, *** p < 0.01, ** p < 0.05, * p < 0.1

### Psychological well-being

**Fig. 1:**
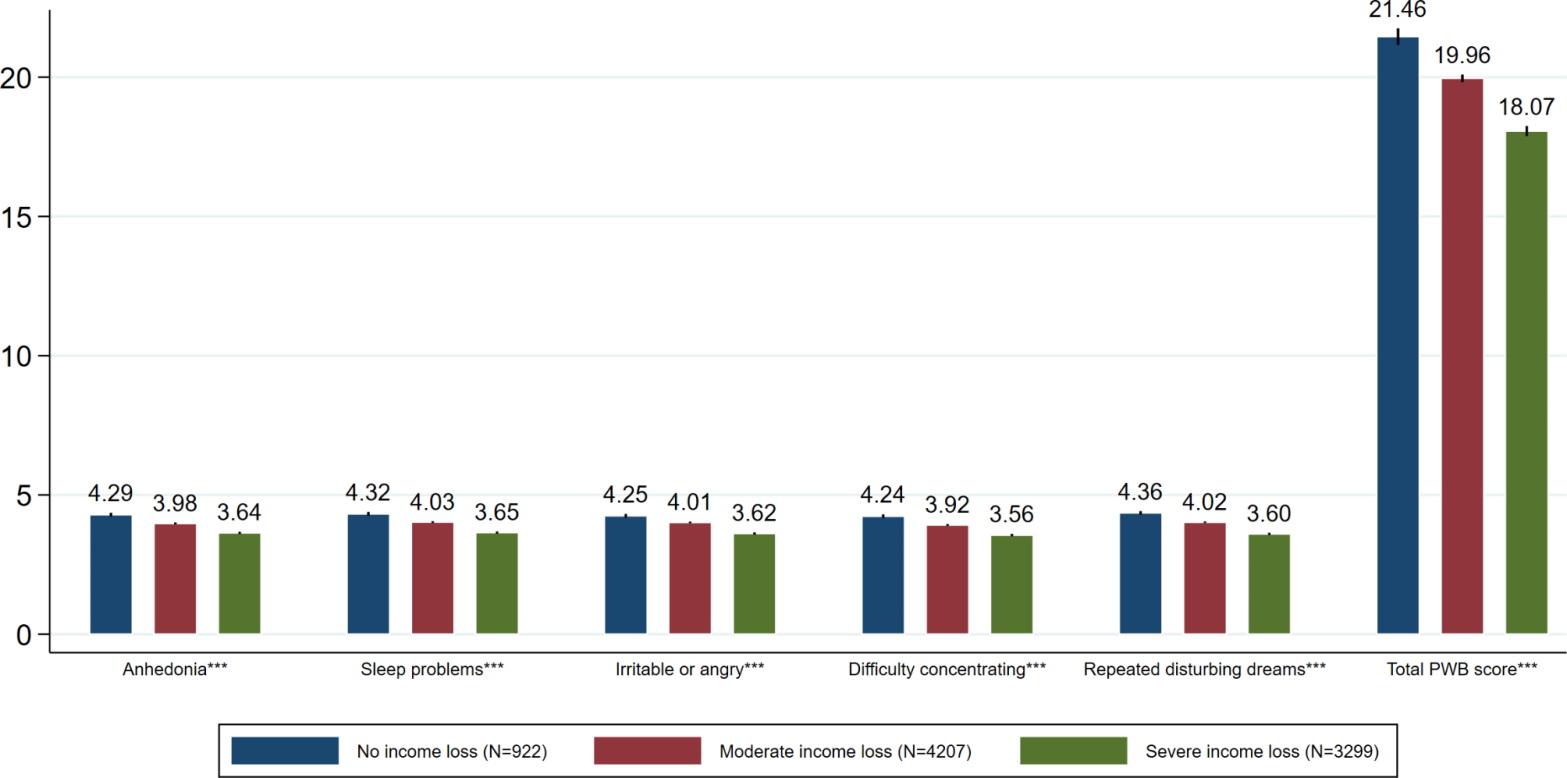
Psychological well-being outcomes between groups with different income shocks among Chinese adults Note: The mean and 95% Confidence Interval of the total PWB score and the score of each item were calculated. The total PWB score ranges from 5 to 25, and the score of each item (i.e. anhedonia, sleep problems, irritable or angry, difficulty concentrating and repeated disturbing dreams) ranges from 1 to 5. A lower score corresponds to poorer psychological well-being PWB: Psychological well-being; *** p < 0.01, ** p < 0.05, * p < 0.1

The average total PWB score for the groups with no income loss, moderate income loss, and severe income loss were 21.46 (SD = 4.56; 95% CI: 21.17–21.76), 19.96 (SD = 4.52; 95% CI: 19.82–20.10) and 18.07 (SD = 5.28; 95% CI: 17.89–18.25), respectively (Fig. 1). Compared to those who had no income loss, those who experienced moderate or severe levels of income loss had lower total PWB scores and lower scores across all domains: anhedonia (3.98 or 3.64 vs. 4.29; P < 0.001), sleep problems (4.03 or 3.65 vs. 4.32; P < 0.001), irritable or angry (4.01 or 3.62 vs. 4.25; P < 0.001), difficulty concentrating (3.92 or 3.56 vs. 4.24; P < 0.001) and repeated disturbing dreams (4.02 or 3.60 vs. 4.36; P < 0.001).

### Income loss and poor psychological well-being

**Fig. 2:**
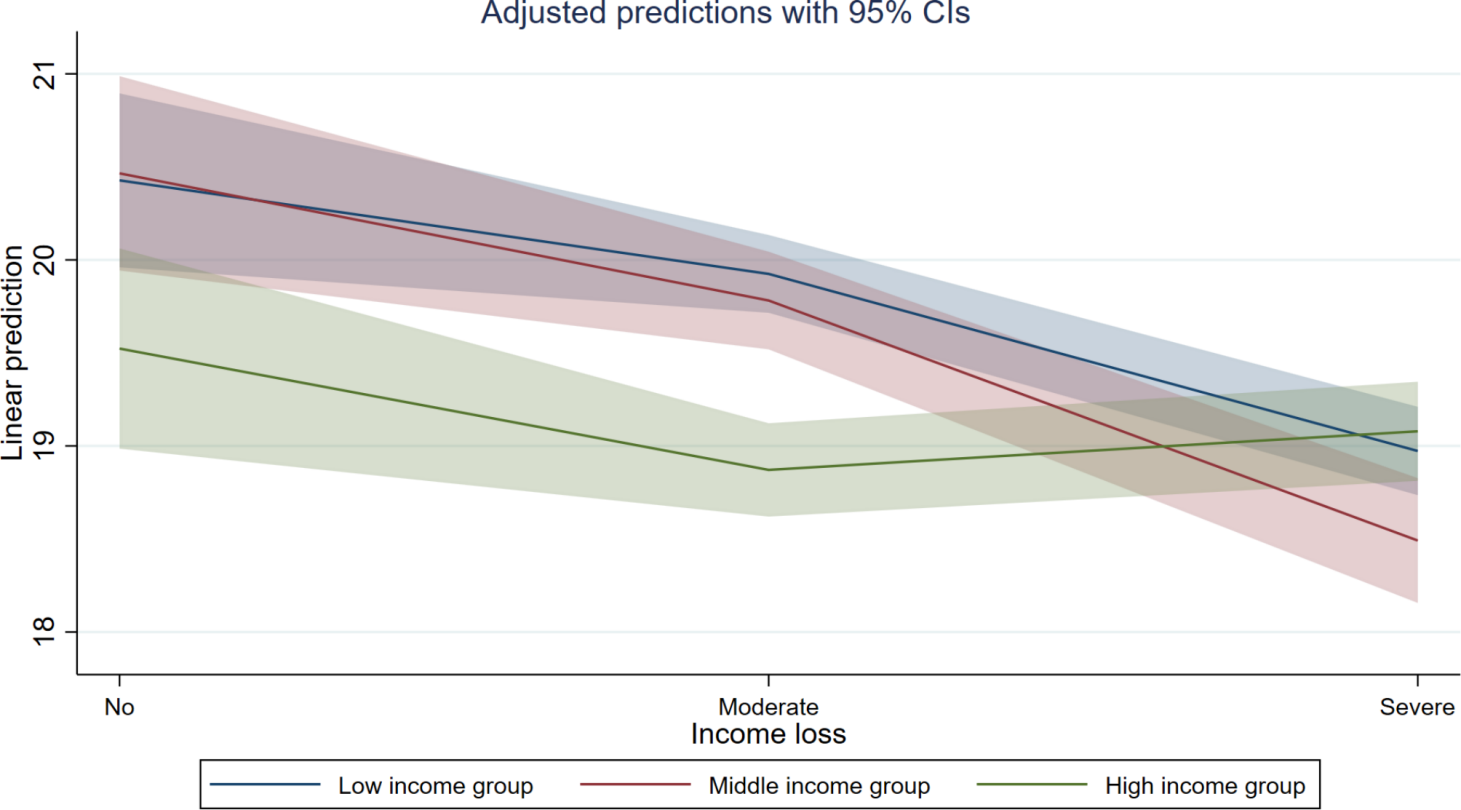
Plots of slopes for the interaction between income loss and pre-pandemic income groups on PWB. Note: All covariates from the set (age, gender, employed status, educational level, self or family member get infected of COVID-19, chronic condition, self-rated health, impact on diet, perceived risk and residential areas) were fixed at their means

The results of hierarchical regression analyses are shown in Table 2. Referring to Model 1, sociodemographic characteristics, health condition, perceived and actual risk of infection, and regional factors accounted for 19.7% of the variance in PWB scores. When income loss was added to the next step in Model 2, an additional 0.6% of variance in PWB scores was captured (adjusted ΔR^2^ = 0.006, P < 0.001), suggesting that income loss was negatively associated with PWB scores (moderate level of income loss: B=-0.603, SE = 0.167; severe level of income loss: B = − 1.261, SE = 0.180). In Model 3, an interaction effect between the degree of income loss and income groups emerged significant. Participants in the middle-income group who had suffered severe income loss scored the lowest on PWB (B = − 1.529, SE = 0.430). The results of the simple slope analyses for the interaction are plotted in Fig. 2. Specifically, the middle income subgroup presented steeper slopes than their respective counterparts, indicating a stronger connection between income loss and poor PWB (moderate vs. no income loss: B = − 0.684, P = 0.020; severe vs. no income loss: B = −-1.974, P < 0.001) (see Supplementary Table 2).

**Table 2:** Associations of income loss with total PWB score among Chinese adults

Variables		Model 1		Model 2		Model 3	
		Coef.	SE	Coef.	SE	Coef.	SE
Income loss	Moderate income loss			-0.603***	0.167	-0.652**	0.303
	Severe income loss			-1.261***	0.180	-0.445	0.308
Interactive items	Moderate income loss×Income (low)					0.150	0.394
	Moderate income loss×Income (middle)					-0.031	0.417
	Severe income loss×Income (low)					-1.010**	0.399
	Severe income loss×Income (middle)					-1.529***	0.430
Age	45–59	1.246***	0.161	1.177***	0.161	1.170***	0.161
	60 or above	1.969***	0.472	1.851***	0.471	1.873***	0.471
Gender	Female	0.288***	0.100	0.238**	0.100	0.239**	0.100
Marital Status	Married or co-habiting	0.682***	0.127	0.656***	0.127	0.644***	0.127
Income level	Income (low)	0.525***	0.117	0.547***	0.117	0.904**	0.359
	Income (middle)	0.371***	0.134	0.328**	0.133	0.942**	0.377
Employment status	Unemployed	-0.332**	0.167	-0.225	0.167	-0.215	0.167
	Non-employed	0.034	0.148	0.026	0.147	0.006	0.147
Education level	Medium education level	-0.112	0.365	-0.081	0.364	-0.117	0.364
	High education level	0.297	0.369	0.293	0.368	0.257	0.367
Chronic medical condition	Having one chronic disease	-1.246***	0.168	-1.239***	0.167	-1.239***	0.167
	Multimorbidity	-2.486***	0.166	-2.476***	0.165	-2.492***	0.165
Self-rated health	Good Self-rated health	-1.109***	0.136	-1.122***	0.135	-1.112***	0.135
	Fair or poor self-rated health	-2.858***	0.300	-2.827***	0.299	-2.828***	0.298
COVID-19 infection	Non-infected COVID-19	1.201***	0.184	1.115***	0.184	1.154***	0.184
Impact on diet	General impact on diet	-1.926***	0.182	-1.768***	0.185	-1.765***	0.184
	High impact on diet	-3.646***	0.184	-3.333***	0.189	-3.334***	0.189
Perceived risks of infection	Perceived medium risk of infection	-0.901***	0.110	-0.824***	0.111	-0.818***	0.110
	Perceived high risk of infection	-1.407***	0.147	-1.149***	0.151	-1.181***	0.151
Risk level of living area	Middle risk areas	-0.077	0.136	-0.069	0.135	-0.067	0.135
	High risk areas	-0.482***	0.139	-0.459***	0.138	-0.458***	0.138
Residential areas	Town	-0.172	0.122	-0.172	0.122	-0.161	0.122
	Rural	-0.088	0.151	-0.046	0.151	-0.036	0.151
	Constant	21.23***	0.467	21.82***	0.477	21.49***	0.521
	Adj R-squared (ΔR-squared)		0.197	0.202(0.006***)		0.205 (0.003***)	

Note: Values were derived from hierarchical multiple regression analysis with total PWB score as dependent variable. Reference levels in the regression is marked in Table 1

Model 1: Control Variables, i.e., age, gender, employed status, educational level, self or family member get infected of COVID-19, chronic condition, self-rated health, impact on diet, perceived risk, residential areas and income level

Model 2: Model 1 variables + income loss

Model 3: Model 1 variables + income loss + interaction terms between income loss and income groups

*** p < 0.01, ** p < 0.05, * p < 0.1

Regression analyses also indicated that younger people, men, singles, high-income groups, unemployed individuals, individuals having one or more chronic medical conditions, those with a history of COVID-19 infection (participants or family members), those with worse self-rated health, individuals whose diets were affected by the COVID-19 pandemic, and those with a perceived medium or high risk of infection and who lived in high-risk areas, were significant associated with worse PWB.

The same results were also observed in each item of PWB (Table 3). Referring to Model 2, income loss explained additional variance in each PWB item: anhedonia (adjusted ΔR^2^ = 0.003, P < 0.001), sleep problems (adjusted ΔR^2^ = 0.004, P < 0.001), irritable or angry (adjusted ΔR^2^ = 0.004, P < 0.001), difficulty concentrating (adjusted ΔR^2^ = 0.003, P < 0.001) and repeated disturbing dreams (adjusted ΔR^2^ = 0.007, P < 0.001). Individuals who suffered from different levels of income shocks differed more widely in their experiences of repeated disturbing dreams as compared to other mental health problems. In Model 3, an interaction effect between the degree of income loss and income groups was also captured in each PWB item. More results of these five terms of PWB including the simple slope analyses for the interaction between income loss and pre-pandemic income groups can be found in Supplementary Tables 2 and Supplementary Fig. 1.

**Table 3:** Associations of income loss with each PWB item among Chinese adults

Variables		Model 1		Model 2		Model 3	
		Coef.	SE	Coef.	SE	Coef.	SE
Anhedonia	Moderate income loss			-0.131***	0.040	-0.119	0.073
	Severe income loss			-0.231***	0.043	-0.052	0.074
	Moderate income loss×Income (low)					0.034	0.095
	Moderate income loss×Income (middle)					-0.084	0.100
	Severe income loss×Income (low)					-0.183*	0.096
	Severe income loss×Income (middle)					-0.387***	0.103
	Adj R-squared (ΔR-squared)	0.140		0.143 (0.003***)		0.145 (0.003***)	
Sleep problems	Moderate income loss			-0.126***	0.040	-0.126*	0.071
	Severe income loss			-0.253***	0.042	-0.074	0.073
	Moderate income loss×Income (low)					-0.011	0.093
	Moderate income loss×Income (middle)					0.012	0.099
	Severe income loss×Income (low)					-0.262***	0.094
	Severe income loss×Income (middle)					-0.276***	0.102
	Adj R-squared (ΔR-squared)	0.154		0.158 (0.004***)		0.161 (0.003***)	
Irritable or angry	Moderate income loss			-0.065	0.040	-0.068	0.072
	Severe income loss			-0.214***	0.043	-0.039	0.073
	Moderate income loss×Income (low)					-0.035	0.093
	Moderate income loss×Income (middle)					0.057	0.099
	Severe income loss×Income (low)					-0.277***	0.095
	Severe income loss×Income (middle)					-0.245**	0.102
	Adj R-squared (ΔR-squared)	0.153		0.156 (0.004***)		0.159 (0.003***)	
Difficulty concentrating	Moderate income loss			-0.121***	0.040	-0.219***	0.073
	Severe income loss			-0.234***	0.043	-0.139*	0.074
	Moderate income loss×Income (low)					0.167*	0.095
	Moderate income loss×Income (middle)					0.103	0.101
	Severe income loss×Income (low)					-0.099	0.096
	Severe income loss×Income (middle)					-0.218**	0.104
	Adj R-squared (ΔR-squared)	0.159		0.162 (0.003***)		0.164 (0.003***)	
Repeated disturbing dreams	Moderate income loss			-0.160***	0.039	-0.120*	0.071
	Severe income loss			-0.329***	0.042	-0.142**	0.072
	Moderate income loss×Income (low)					-0.006	0.092
	Moderate income loss×Income (middle)					-0.118	0.097
	Severe income loss×Income (low)					-0.189**	0.093
	Severe income loss×Income (middle)					-0.404***	0.100
	Adj R-squared (ΔR-squared)	0.145		0.153 (0.007***)		0.155 (0.003***)	

Note: All Values were derived from hierarchical multiple regression analysis with the score of each PWB item (anhedonia, sleep problems, irritable or angry, difficulty concentrating and repeated disturbing dreams) as dependent variable

Model 1: Control Variables, i.e., age, gender, marital Status, employed status, educational level, self or family member get infected of COVID-19, chronic condition, self-rated health, impact on diet, perceived risk, residential areas and income level

Model 2: Model 1 variables + income loss

Model 3: Model 1 variables + income loss + interaction terms between income loss and income groups

*** p < 0.01, ** p < 0.05, * p < 0.1

The sensitivity analysis yielded results that are presented in Supplementary Tables 3 and Table 4. In the hierarchical multiple linear regressions containing only non-employed samples, the association between household income loss and PWB remained robust (see Supplementary Table 3). Among adult students, the correlation between household income loss and PWB decreased, but remained significant (severe level of income loss: B = − 0.850, SE = 0.451, P = 0.060 in Model 2; severe level of income loss × middle income group: B = − 3.027, SE = 1.287, P = 0.019 in Model 3) (see Supplementary Table 4).

## Discussion

In the early stages of the COVID-19 pandemic, stringent anti-epidemic policies proved very effective in controlling the spread of infection, but they upset the normality of daily life and work, causing a great impact on many industries, such as the catering, tourism and transportation industries, and forcing employees to take pay cuts or even lose their jobs. Many individuals were threatened with both health problems and income loss during the early COVID-19 pandemic. This study found that, during the early pandemic, about 90% of Chinese adults suffered a moderate or severe loss of household income, and income loss had detrimental psychological consequences. The effect of income shock on PWB varied by income level in the year before the pandemic.

Moderate or severe income loss during the pandemic was associated with poor PWB and mental health problems such as anhedonia, sleep problems, irritability or anger, difficulty with concentration and repeated disturbing dreams related to COVID-19. The association remained significant even after adjusting for individual and environmental confounders. These findings are in line with previous COVID-19-related studies [5, 27] and previous findings on events unrelated to COVID-19 (such as recession, stock market crash) which similarly found an association between unemployment, financial difficulties, wealth loss and poor subjective mental health [45–47]. For example, economic stress was specifically associated with seeking mental health support due to depression in past recessions [48]. Economic distress related to natural disaster was also found to increase the risk of depression [49]. Evidence from the field of social epidemiology likewise supports a relationship between economic stress and health [50]. Based on the resource-oriented model of stress, stress is triggered when individuals perceive a threat or loss to resources, which encompass anything of value to them [51, 52]. Income loss not only hinders future planning but also reduces one’s ability to purchase necessities to meet current needs, such as food, thereby increasing the risk of food insecurity, unhealthy lifestyles, abnormal household dynamics, and health care-seeking behavior [20, 53], all of which could lead to psychological problems. In addition, our study revealed that the proportion of individuals experiencing a decline in household income was 90%, a figure that may appear elevated compared to the rates reported in other countries or regions as cited in the introduction [5–9]. The inconsistency may be caused by difference in the study sample (e.g., exclusively workers or encompassing all adults), different definition of income loss (e.g., personal income loss versus household income loss), different stages of the pandemic, and varying degrees of stringency in pandemic prevention and control measures across different nations or regions. Consequently, it is imperative to exercise caution when drawing comparisons between these findings.

Our study also extended the existing literature by examining the interaction effect between level of income loss and past income status on psychological well-being [30]. Specifically, individuals in the middle-income group who experienced severe loss of income were more likely to report poor psychological well-being than other income groups. Whether a particular individual or group is at high risk of maladaptation following an income shock depends on the extent to which the shock was anticipated, its persistence, and the ability of the household to buffer these shocks [54]. The habituation theory suggests that an individual always takes into account their past income status, and loss has a negative effect on well-being [55]. Compared with the health of the wealthy, lower income groups are more vulnerable to environmental disadvantages [56]. The literature suggests that middle-income groups suffer increased stress levels following economic shocks [57]. Compared with higher income individuals, people in the middle- or lower-income groups may be unable to buffer such income shocks or have limited resources to adapt to income fluctuations and support their current standards of living, which could partly explain our findings. The mechanisms underlying these differences in household income gradients were complex and involved a plethora of confounding factors, such as coping resources, cognitive appraisal, coping strategies, human capital [58, 59]. In general, regardless of household income or household position on the general income distribution, income stability is essential for PWB.

Moreover, our results showed that older adults were less likely to experience the negative effects of early COVID-19 pandemic on psychological well-being compared to younger individuals. Research examining age differences in mental health during the COVID-19 pandemic has been inconclusive. Some research showed that anxiety levels during the pandemic were positively associated with age [60], while others found a negative association [61, 62] or few associations [63]. Previous research showed that mental toughness increases with age and experience, with mental toughness being negatively correlated with depression, anxiety, and stress [64]. Our results showed that poor psychological well-being was associated with poor physical health (i.e., one or more chronic medical condition or having poor self-rated health). The results we obtained are also consistent with studies showing that health status is closely related to psychosocial well-being [65]. The perceived and actual risks related to COVID were found to be critical indicators for psychosocial well-being, which was identified in other studies [23, 66]; people with a higher risk perception of the pandemic were more likely to panic and respond unfavorably [23].

Given the health-economic trade-offs, our results have significant implications for the development of COVID-19-related social protection policies in future pandemic events. Given that individuals who experienced high levels of income loss were at increased risk of psychological problems, particularly those with middle income before the pandemic, our study highlights the necessity of paying attention to mental health issues during the pandemic, and the importance of maintaining a balance between performing necessary pandemic preventive strategies and minimizing individual income shocks, especially for economically vulnerable individuals. Both the specialized outpatient mental health care institutions and primary care institutions should strengthen services related to psychological counseling and humanistic care for the public. In addition, strong informal social networks that can provide individuals with solace, security, assurance, and support should be substantially strengthened to help individuals adopt more optimal coping approaches and therefore alleviate the negative impact of income shocks on mental health [67, 68]. Strategies aimed at addressing the negative consequences of income loss include problem-focused coping, which involves actions such as reducing expenses, seeking alternative employment opportunities, engaging in self-directed learning, and acquiring additional training either online or offline to improve one’s job prospects. Emotion-focused coping, such as self-soothing (e.g., relaxation, seeking emotional support), expression of negative emotion (e.g., confiding in others), and considering the problem more calmly, were suggested for minimizing distress triggered by stressors [51]. Indubitably, there is a pressing need to strengthen mental health care systems in China.

This study has several limitations. First, due to the cross-sectional nature of this study, we are unable to draw definite conclusions about causation. Despite efforts to control for confounders and conduct sensitivity analysis, it was challenging to entirely eliminate reverse-causation bias due to the potential influence of psychological well-being on income loss. However, the results of sensitivity analyses conducted on the subgroup of non-employed individuals (i.e., adult students and retired workers) indicated the robustness of the findings. It is improbable that poor PWB among non-employed individuals affects household income loss. But instead, household finance-related psychological distress may have a spillover effect, affecting other family members [59, 69]. Moreover, in the context of the strict prevention and control of COVID-19 epidemic, the free employment and working environment were broken. It is important to note that the survey was conducted during a phase when China was gradually resuming work and production. Consequently, the influence of poor mental health on employment and work performance was limited within the relatively brief period of returning to work. Thus, we believed the possibility of reverse-causation bias was minimal. Second, the participants were recruited using the snowball sampling approach through social media. Potential biases related to the use of online surveys for data collection during the COVID-19 pandemic should be considered [70], since this approach attracts volunteers who are interested in the topic and can access the internet. However, we attempted to overcome this limitation by controlling for confounding factors. Third, given that the degree of income loss was self-reported, participants could have under- or overestimated their income loss. The perception of income loss severity could be influenced by previous income status, “financial buffering” capacity (i.e., savings and other financial resources) and environmental factors [5]. Future studies should employ precise measurements of actual income loss.

This study has several strengths. First, our results extended the extant literature by probing the effect of income shocks on psychological well-being. Prior to this study, there was substantial evidence pointing to the negative impacts of COVID-19 and its transmission preventive strategies on mental health, but little attention had been paid to the mental health consequences of COVID-19-related income shocks. Second, PWB was used as a health outcome rather than an objective measure of health. Declines in physical health as a result of COVID-19-related stress are unlikely to become evident in the short run. Biological pathways also suggest that psychological health may be affected before physical health [55]. Therefore, in the absence of biomarkers, subjective assessments of health are useful in assessing early changes in health status and could help to increase the reliability of the results. Third, in contrast to previous studies mostly focusing on the impact of income shocks or unemployment on psychological well-being in the context of a recession, our study contributed to the existing knowledge base by examining the relation between income loss and psychological well-being in the pandemic context. The two contexts (recession vs. pandemic) are starkly different because a pandemic poses the double risks of physical health deterioration and income loss. Furthermore, our findings inform future public policy by emphasizing the importance of income security to mental health.

## Conclusions

This study sheds light on the negative impacts of income loss during the pandemic on psychological well-being and the interaction between extent of income loss and previous income levels. Our study highlights the necessity of paying attention to the psychological well-being of the population during a pandemic, especially individuals suffering from income shocks. There is a need to strengthen primary care to ensure that counseling and humanistic care services are available to the public. There is also a need to establish strong informal social networks to help individuals achieve a more optimal coping approach to alleviate the negative impacts of the pandemic.

### Electronic supplementary material

Below is the link to the electronic supplementary material.

Supplementary Material 1

## Data Availability

The datasets presented in this article are not readily available because ethics restrictions. Requests to access the datasets should be directed to youfawang@gmail.com.

## List of Abbreviations

PWB: Psychological well-being
COVID-19: Coronavirus disease 2019
