# Supplementary material for: Income loss and subsequent poor psychological well-being among the Chinese population during the early COVID-19 pandemic
Source: Int J Equity Health. 2023 Oct 17;22:219. doi: 10.1186/s12939-023-02022-1 (PMC10583462; doi:10.1186/s12939-023-02022-1)
Supplement: Supplementary file 1 — Supplementary Material 1 [file 12939_2023_2022_MOESM1_ESM.docx]

Supplemental Table 1 Univariate linear regression models for confounding factors

| Variables |  | Coef. | SE |
| --- | --- | --- | --- |
| Age (years) | 45-59 | 1.646*** | 0.171 |
|  | 60 or above | 2.076*** | 0.508 |
| Gender | Female | 0.776*** | 0.109 |
| Marital Status | Married or co-habiting | 0.721*** | 0.116 |
| Employment status | Unemployed | -0.779*** | 0.175 |
|  | Non-employed | -0.269** | 0.136 |
| Education level | Medium education level | 1.104*** | 0.401 |
|  | High education level | 1.663*** | 0.399 |
| Chronic medical condition | Having one chronic disease | -1.734*** | 0.176 |
|  | Multimorbidity | -3.859*** | 0.168 |
| Self-rated health | Good Self-rated health | -1.113*** | 0.147 |
|  | Fair or poor self-rated health | -3.625*** | 0.328 |
| COVID-19 infection | Non-infected COVID-19 | 3.241*** | 0.183 |
| Impact on diet | General impact on diet | -2.157*** | 0.192 |
|  | High impact on diet | -4.710*** | 0.190 |
| Perceived risks of infection | Perceived medium risk | -1.560*** | 0.117 |
|  | Perceived high risk | -3.209*** | 0.145 |
| Risk level of living area | Middle risk areas | 0.101 | 0.150 |
|  | High risk areas | -0.760*** | 0.148 |
| Residential areas | Town | 0.0507 | 0.130 |
|  | Rural | -0.144 | 0.156 |
| Income level | Income (low) | 1.091*** | 0.126 |
|  | Income (middle) | 0.949*** | 0.143 |

Note: Values were derived from univariate linear regression analysis with total PWB score as dependent variable. Reference levels in the regression is marked in Table 1.

*** p<0.01, ** p<0.05, * p<0.1.

Supplemental Table 2 Results of simple slope test for the interaction between income loss and pre-pandemic income groups on PWB and each PWB item

| Income loss | Income groups | Total PWB score  Coef. (SE) | Anhedonia  Coef. (SE) | Sleep problems  Coef. (SE) | Irritable or angry  Coef. (SE) | Difficulty concentrating  Coef. (SE) | Repeated disturbing dreams  Coef. (SE) |
| --- | --- | --- | --- | --- | --- | --- | --- |
| Moderate vs no income loss | Low | -0.503 | -0.084 | -0.136 | -0.104 | -0.052 | -0.126 |
|  |  | (0.259) * | -0.062 | (0.061) ** | (0.061) * | -0.062 | (0.060) ** |
| Moderate vs no income loss | Middle | -0.684 | -0.203 | -0.114 | -0.012 | -0.117 | -0.239 |
|  |  | (0.295) ** | (0.071) *** | -0.07 | -0.07 | -0.071 | (0.069) *** |
| Moderate vs no income loss | High | -0.652 | -0.119 | -0.126 | -0.068 | -0.219 | -0.120 |
|  |  | (0.303) ** | -0.073 | (0.071) * | -0.072 | (0.073) *** | (0.070) * |
| Severe vs no income loss | Low | -1.454 | -0.235 | -0.335 | -0.315 | -0.238 | -0.331 |
|  |  | (0.270) *** | (0.065) *** | (0.064) *** | (0.064) *** | (0.065) *** | (0.063) *** |
| Severe vs no income loss | Middle | -1.974 | -0.439 | -0.349 | -0.283 | -0.357 | -0.546 |
|  |  | (0.318) *** | (0.076) *** | (0.075) *** | (0.075) *** | (0.077) *** | (0.074) *** |
| Severe vs no income loss | High | -0.445 | -0.052 | -0.074 | -0.038 | -0.139 | -0.142 |
|  |  | -0.308 | -0.074 | -0.073 | -0.073 | (0.074) * | (0.072) ** |

Note: Other covariates from the set (age, gender, employed status, educational level, self or family member get infected of COVID-19, chronic condition, self-rated health, impact on diet, perceived risk and residential areas) were controlled in the analysis.

*** p<0.01, ** p<0.05, * p<0.1.

| 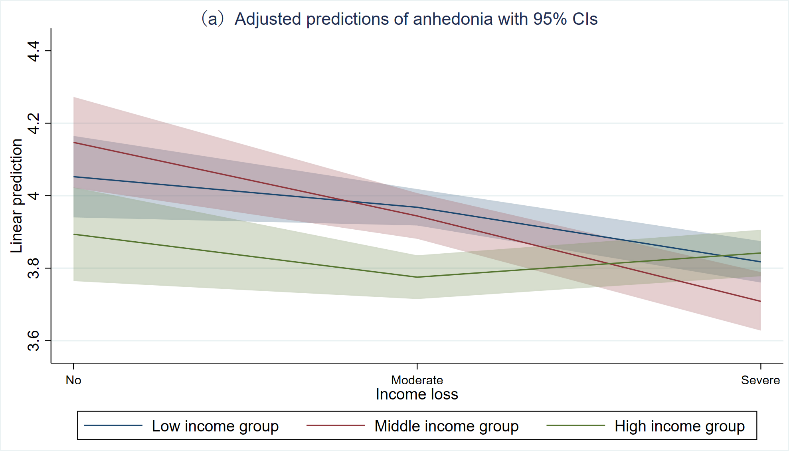 | 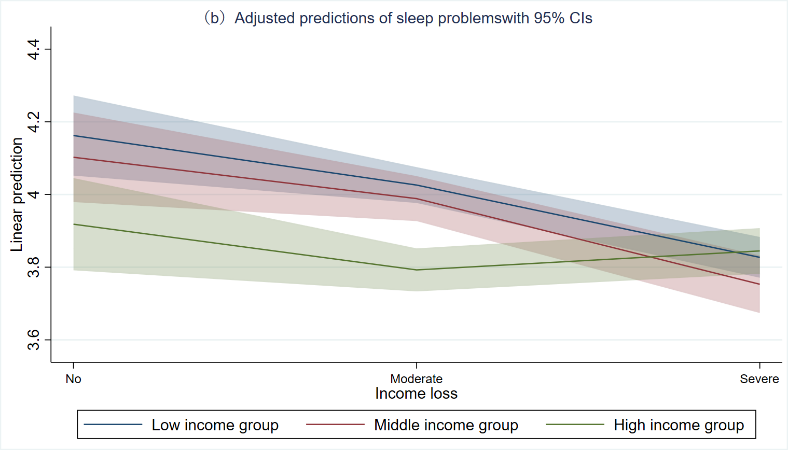 |
| --- | --- |
| 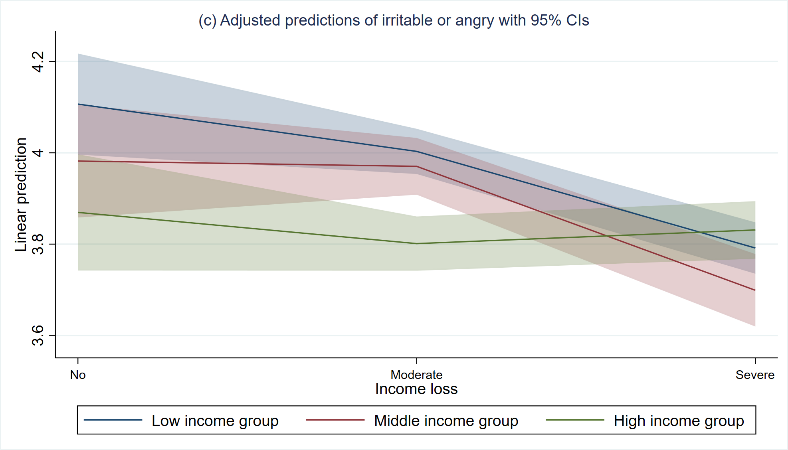 | 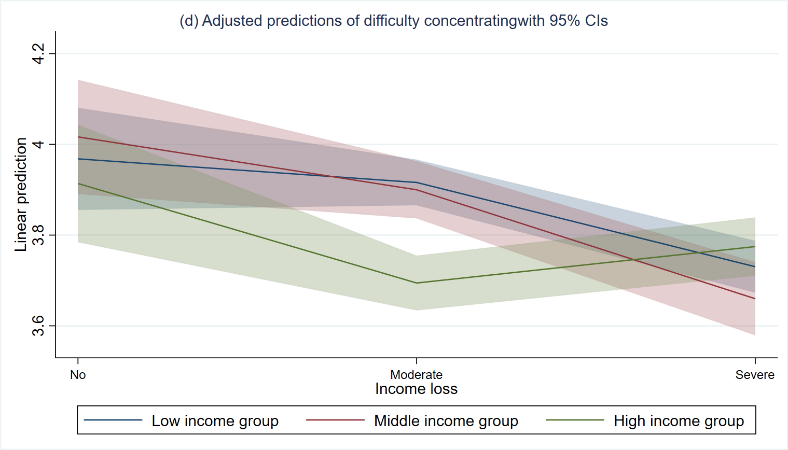 |
| 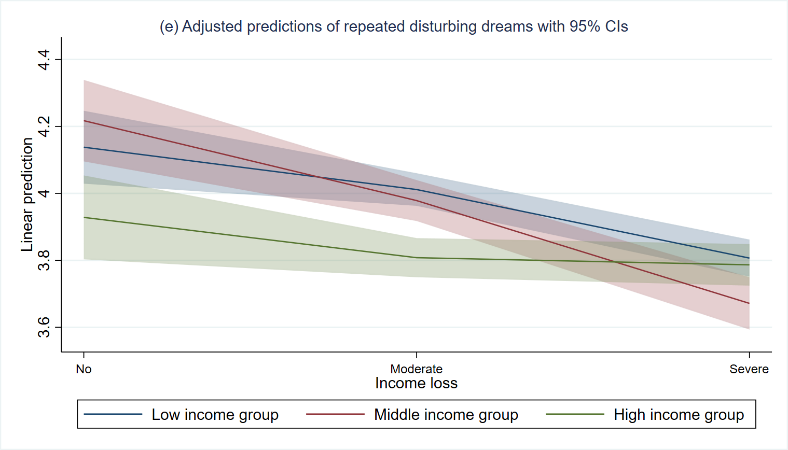 |  |

Supplemental Figure 1 Plots of slopes for the interaction between income loss and pre-pandemic income groups on each PWB item.

Note: All covariates from the set (age, gender, employed status, educational level, self or family member get infected of COVID-19, chronic condition, self-rated health, impact on diet, perceived risk and residential areas) were fixed at their means in the analysis.

Supplemental Table 3 Sensitivity analysis: Associations of income loss with total PWB score among non-employed group.

| Variables |  | Model 1 | |  | Model 2 | |  | Model 3 | |
| --- | --- | --- | --- | --- | --- | --- | --- | --- | --- |
|  |  | Coef. | SE |  | Coef. | SE |  | Coef. | SE |
| Income loss | Moderate income loss |  |  |  | -0.567 | 0.363 |  | -0.714 | 0.636 |
|  | Severe income loss |  |  |  | -1.048*** | 0.382 |  | 0.0124 | 0.635 |
| Interactive items | Moderate income loss×Income (low) |  |  |  |  |  |  | 0.141 | 0.801 |
|  | Moderate income loss×Income (middle) |  |  |  |  |  |  | 0.274 | 1.050 |
|  | Severe income loss×Income (low) |  |  |  |  |  |  | -1.323 | 0.808 |
|  | Severe income loss×Income (middle) |  |  |  |  |  |  | -2.709** | 1.096 |
| Age (years) | 45-59 | 2.320*** | 0.512 |  | 2.289*** | 0.511 |  | 2.276*** | 0.510 |
|  | 60 or above | 3.608*** | 0.636 |  | 3.472*** | 0.636 |  | 3.418*** | 0.637 |
| Gender | Female | 0.530** | 0.231 |  | 0.467** | 0.232 |  | 0.471** | 0.231 |
| Marital Status | Married or co-habiting | -0.342 | 0.360 |  | -0.329 | 0.360 |  | -0.335 | 0.359 |
| Income level | Income (low) | 0.495* | 0.254 |  | 0.501** | 0.253 |  | 1.025 | 0.723 |
|  | Income (middle) | 0.793** | 0.360 |  | 0.730** | 0.361 |  | 1.627* | 0.925 |
| Education level | Medium education level | -0.539 | 0.697 |  | -0.551 | 0.696 |  | -0.535 | 0.694 |
|  | High education level | -0.0345 | 0.698 |  | -0.0362 | 0.697 |  | -0.0856 | 0.694 |
| Chronic medical condition | Having one chronic disease | -1.655*** | 0.395 |  | -1.673*** | 0.394 |  | -1.687*** | 0.393 |
|  | Multimorbidity | -2.777*** | 0.392 |  | -2.476*** | 0.392 |  | 2.492*** | 0.391 |
| Self-rated health | Good Self-rated health | -1.118*** | 0.290 |  | -1.131*** | 0.290 |  | -1.103*** | 0.289 |
|  | Fair or poor self-rated health | -3.323*** | 0.548 |  | -3.287*** | 0.547 |  | -3.190*** | 0.546 |
| COVID-19 infection | Non-infected COVID-19 | 0.532 | 0.421 |  | 0.502 | 0.421 |  | 0.610 | 0.421 |
| Impact on diet | General impact on diet | -1.382*** | 0.384 |  | -1.292*** | 0.387 |  | -1.342*** | 0.386 |
|  | High impact on diet | -2.598*** | 0.392 |  | -2.393*** | 0.398 |  | -2.478*** | 0.398 |
| Perceived risks | Perceived medium risk of infection | -0.768*** | 0.240 |  | -0.728*** | 0.241 |  | -0.692*** | 0.240 |
|  | Perceived high risk of infection | -1.337*** | 0.334 |  | -1.177*** | 0.339 |  | -1.201*** | 0.338 |
| Risk level of living area | Middle risk areas | -0.395 | 0.281 |  | -0.365 | 0.281 |  | -0.422 | 0.280 |
|  | High risk areas | -0.728** | 0.294 |  | -0.698** | 0.294 |  | -0.700** | 0.293 |
| Residential areas | Town | -0.250 | 0.276 |  | -0.218 | 0.276 |  | -0.241 | 0.275 |
|  | Rural | 0.215 | 0.275 |  | 0.291 | 0.276 |  | 0.312 | 0.275 |
|  | Constant | 21.58*** | 0.866 |  | 22.12*** | 0.892 |  | 21.69*** | 0.982 |
|  | Adj R-squared (ΔR-squared) | 0.190 | |  | 0.193 (0.003**) | |  | 0.199(0.006***) | |

Note: Values were derived from hierarchical multiple regression analysis with total PWB score as dependent variable. Reference levels in the regression is marked in Table 1.

Model 1: Control Variables, i.e., age, gender, employed status, educational level, self or family member get infected of COVID-19, chronic condition, self-rated health, impact on diet, perceived risk, residential areas and income level.

Model 2: Model 1 variables + income loss.

Model 3: Model 1 variables + income loss +interaction terms between income loss and income groups.

*** p<0.01, ** p<0.05, * p<0.1.

Supplemental Table 4 Sensitivity analysis: Associations of income loss with total PWB score among students aged 18 years or above.

| Variables |  | Model 1 | |  | Model 2 | |  | Model 3 | |
| --- | --- | --- | --- | --- | --- | --- | --- | --- | --- |
|  |  | Coef. | SE |  | Coef. | SE |  | Coef. | SE |
| Income loss | Moderate income loss |  |  |  | -0.312 | 0.436 |  | -0.340 | 0.798 |
|  | Severe income loss |  |  |  | -0.850* | 0.451 |  | 0.527 | 0.788 |
| Interactive items | Moderate income loss×Income (low) |  |  |  |  |  |  | -0.135 | 0.989 |
|  | Moderate income loss×Income (middle) |  |  |  |  |  |  | 0.331 | 1.253 |
|  | Severe income loss×Income (low) |  |  |  |  |  |  | -1.806* | 0.992 |
|  | Severe income loss×Income (middle) |  |  |  |  |  |  | -3.027** | 1.287 |
| Age (years) | 45-59 | -2.139 | 1.737 |  | -2.081 | 1.736 |  | -2.138 | 1.729 |
| Gender | Female | 0.492* | 0.251 |  | 0.430* | 0.252 |  | 0.411 | 0.252 |
| Marital Status | Married or co-habiting | -0.787* | 0.436 |  | -0.739* | 0.436 |  | -0.779* | 0.435 |
| Income level | Income (low) | 0.485* | 0.283 |  | 0.480* | 0.283 |  | 1.392 | 0.906 |
|  | Income (middle) | 0.905** | 0.398 |  | 0.834** | 0.398 |  | 1.903* | 1.120 |
| Education level | Medium education level | -1.563* | 0.819 |  | -1.563* | 0.818 |  | -1.624** | 0.815 |
|  | High education level | -0.990 | 0.816 |  | -0.988 | 0.815 |  | -1.085 | 0.811 |
| Chronic medical condition | Having one chronic disease | -2.009*** | 0.501 |  | -1.239*** | 0.500 |  | -1.239*** | 0.498 |
|  | Multimorbidity | -3.210*** | 0.465 |  | -3.192*** | 0.465 |  | -3.199*** | 0.465 |
| Self-rated health | Good Self-rated health | -1.135*** | 0.325 |  | -1.152*** | 0.325 |  | -1.119*** | 0.324 |
|  | Fair or poor self-rated health | -3.169*** | 0.621 |  | -3.144*** | 0.620 |  | -3.077*** | 0.618 |
| COVID-19 infection | Non-infected COVID-19 | 0.220 | 0.457 |  | 0.174 | 0.458 |  | 0.278 | 0.457 |
| Impact on diet | General impact on diet | -1.261*** | 0.437 |  | -1.222*** | 0.441 |  | -1.241*** | 0.440 |
|  | High impact on diet | -2.418*** | 0.444 |  | -3.333*** | 0.451 |  | -3.334*** | 0.449 |
| Perceived risks | Perceived medium risk of infection | -0.771*** | 0.266 |  | -0.744*** | 0.266 |  | -0.692*** | 0.266 |
|  | Perceived high risk of infection | -1.350*** | 0.366 |  | -1.195*** | 0.371 |  | -1.224*** | 0.370 |
| Risk level of living area | Middle risk areas | -0.539* | 0.311 |  | -0.526* | 0.310 |  | -0.590* | 0.309 |
|  | High risk areas | -0.770** | 0.325 |  | -0.764** | 0.325 |  | -0.766** | 0.324 |
| Residential areas | Town | -0.404 | 0.306 |  | -0.376 | 0.306 |  | -0.401 | 0.305 |
|  | Rural | 0.180 | 0.293 |  | 0.246 | 0.295 |  | 0.282 | 0.294 |
|  | Constant | 22.88*** | 0.994 |  | 23.30*** | 1.030 |  | 22.67*** | 1.156 |
|  | Adj R-squared (ΔR-squared) | 0.174 | |  | 0.176(0.002*) | |  | 0.184(0.008***) | |

Note: Values were derived from hierarchical multiple regression analysis with total PWB score as dependent variable. Reference levels in the regression is marked in Table 1.

Model 1: Control Variables, i.e., age, gender, employed status, educational level, self or family member get infected of COVID-19, chronic condition, self-rated health, impact on diet, perceived risk, residential areas and income level.

Model 2: Model 1 variables + income loss.

Model 3: Model 1 variables + income loss +interaction terms between income loss and income groups.

*** p<0.01, ** p<0.05, * p<0.1.
